# Assessment of Occlusal Function in a Patient with an Angle Class I Spaced Dental Arch with Periodontal Disease Using a Brux Checker

**DOI:** 10.1155/2018/3876297

**Published:** 2018-01-31

**Authors:** Ayako Taira, Shiho Odawara, Shuntaro Sugihara, Kenichi Sasaguri

**Affiliations:** ^1^Department of Dentistry, Oral and Maxillofacial Surgery, Jichi Medical University, 3311-1 Yakushiji, Shimotsuke, Tochigi 329-0498, Japan; ^2^Division of Orthodontics, Department of Oral Science, Kanagawa Dental University, 82 Inaoka-cho, Yokosuka, Kanagawa 238-8580, Japan; ^3^Division of Periodontology, Department of Oral Function and Restoration, Kanagawa Dental University, 82 Inaoka-cho, Yokosuka, Kanagawa 238-8580, Japan

## Abstract

Comprehensive and appropriate occlusion reconstruction therapy is necessary for orthodontic treatment of adult patients with malocclusion with periodontal disease associated with occlusal trauma. We report the case of a patient with extensive moderate chronic periodontitis associated with occlusal trauma. The patient was diagnosed with extensive moderate chronic periodontitis associated with occlusal trauma and underwent thorough treatment for periodontal disease, oral management, and 20 months of orthodontic therapy. Moreover, reconstructed occlusion was performed to evaluate occlusal trauma for visualization using Brux Checker (BC) analysis before and after active orthodontic treatment. The patient acquired stable anterior guidance and a functional occlusal relationship. BC findings revealed weakening of the functional contact between the lateral occlusal force of the dentition and the front teeth and alveolar bone regeneration. The laminar dura became clearer, and the periodontal tissue improved. Our results suggest that assessment of occlusion function using BC analysis and periodontal examination was effective in enabling occlusal treatment goal clarification through orthodontic treatment in case of periodontal disease associated with occlusal trauma.

## 1. Introduction

Orthodontic treatment for adult patients with malocclusion complicated by periodontal disease includes comprehensive and appropriate occlusal reconstruction and ongoing oral management during treatment for periodontal disease and the orthodontic process before starting active treatment [1–3]. In particular, when implementing occlusal reconstruction using orthodontic treatment in patients with suspected occlusal trauma, it is possible to achieve appropriate disclusion in the molar region by providing correct anterior guidance, and it is important to weaken the lateral occlusal force. In this case, the patient was suspected to have extensive, moderate chronic periodontitis [4] associated with occlusal trauma; therefore, a periodontal disease specialist provided adequate treatment for periodontal disease and oral management before orthodontic treatment commenced. When occlusal reconstruction was performed with orthodontic treatment, occlusion was confirmed at the dental chair side, and night-time parafunction was assessed using a Brux Checker (BC; Rocky Mountain Morita Corporation, Tokyo, Japan) [5, 6]. Further, after the patient had used a retainer for 2 years, she underwent coronally advanced flap repositioning surgery with a connective tissue graft for root coverage of the bilateral maxillary lateral incisors to enable cosmetic and hygiene restoration for gingival recession on the labial side of the tooth cervix of the maxillary bilateral incisors. The patient has made a good recovery.

## 2. Case Presentation

### 2.1. Diagnosis

**Figure 1:**
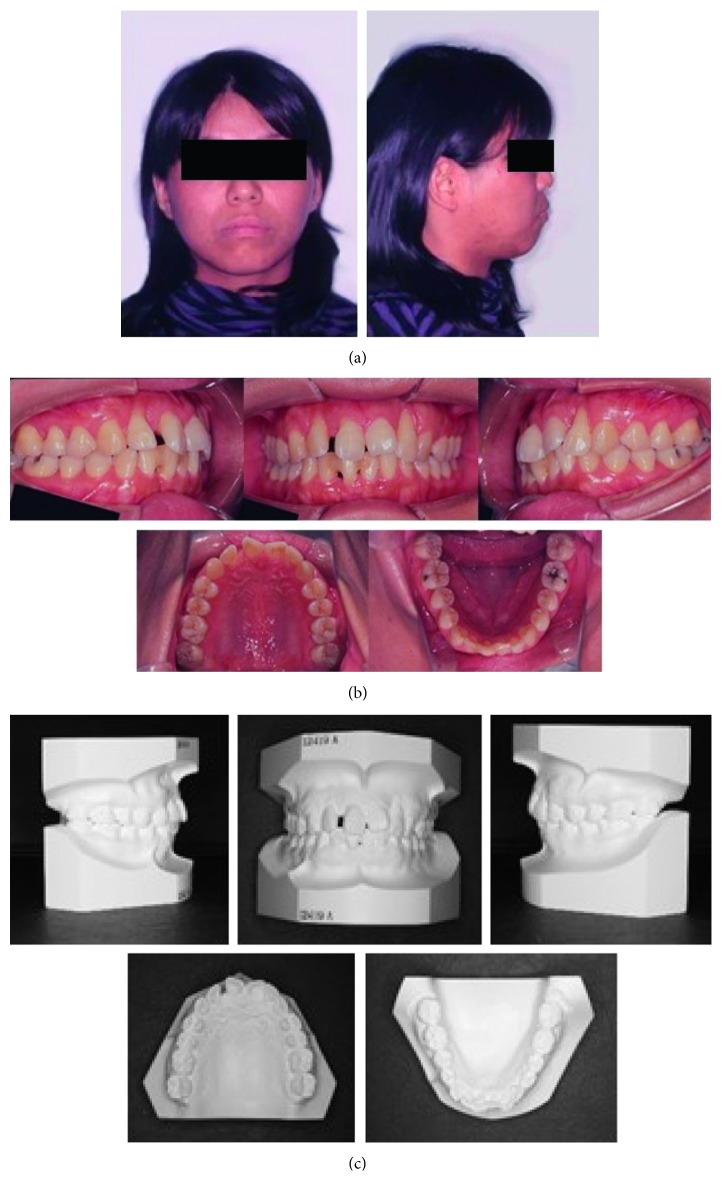
Pre-orthodontic treatment facial (a) and intraoral photographs (b) and dental casts (c) are shown.

**Figure 2:**
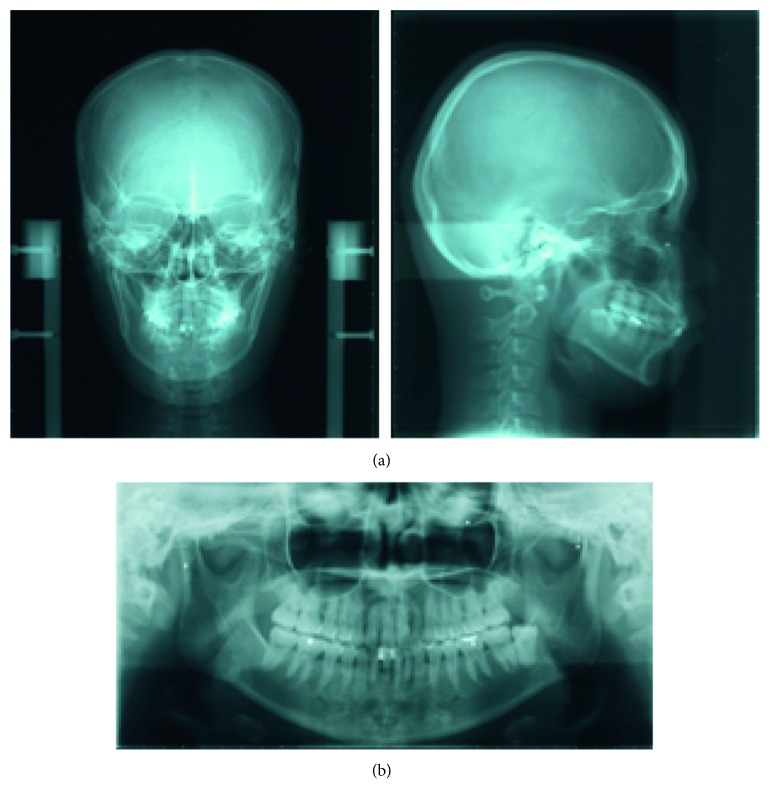
Pre-orthodontic treatment cephalograms (a) and panoramic radiograph (b) are shown.

The patient was a woman aged 30 years and 2 months at the initial consultation. She visited the hospital with a chief complaint of severe pain in her maxillary and mandibular front teeth and a spaced dental arch. Her face was bilaterally symmetric, and she had a convex facial profile (Figure 1). The anterior occlusal relationship included an overjet of 2 mm and an overbite of 4 mm; the molar occlusal relationship was bilateral Angle Class I, and the arch length discrepancy was +2 mm for both the maxilla and mandible. The lateral dentition had relatively good occlusion, but the bilateral maxillary central incisors showed mesial rotation, and her previous dentist had joined the mandibular front teeth with resin, presumably to prevent tooth mobility (Figures 1 and 1). Cephalometric analysis showed the following: ANB, 6; FMA, 30; U1-SN, 106; and IMPA, 99 (Figure 2 and Table 1). Therefore, the patient's facial type demonstrated a mesofacial pattern, and she was diagnosed orthodontically with an Angle Class I spaced dental arch.

**Figure 3:**
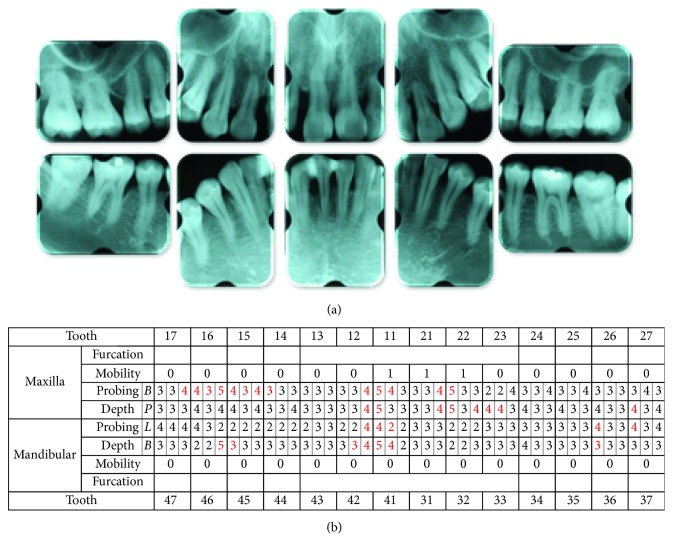
Pre-orthodontic treatment dental radiographs (a) and periodontal examinations (b) are shown. The red color indicates bleeding on probing.

**Figure 4:**
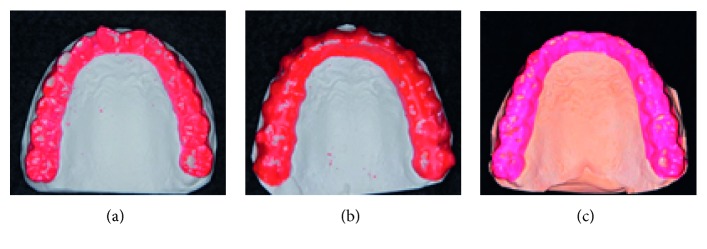
Brux Checker findings. (a) Before orthodontic treatment, (b) just before removal of the orthodontic appliance, and (c) at 2 years after retention.

The radiographic findings indicated vertical bone resorption in the mesial portions of the right maxillary and mandibular first molars, and the maxillary and mandibular front teeth showed high-grade bone resorption (Figures 2 and 3). On the periodontal disease chart, there were swelling of the gingiva in the same location and a periodontal pocket exceeding 4 mm. Bleeding on probing was observed (Figure 3). The front teeth of the lower jaw had been joined with resin at another hospital to prevent tooth mobility. There was also moderate loss of the mesial interdental papilla in the gingival recession area of the bilateral maxillary lateral incisors (Figures 1 and 3). Dental findings showed a reduction in the mesial bone level; therefore, the patient was diagnosed as Class III according to Miller's classification of gingival recession [7]. In this case, given that periodontal lesions were found in the maxillary and mandibular teeth and molar areas, the patient was diagnosed with malocclusion associated with extensive moderate chronic periodontitis. Further, when we examined night-time parafunction using a BC [5, 6], strong functional contact was noted on the marginal ridges on the mesial side of the bilateral maxillary central incisors and the incisal edges of the bilateral lateral incisors, as well as the right canine, first premolar, and first molar (Figure 4). These locations generally correlated with the locations of the patient's symptoms of periodontal disease according to the periodontal disease chart (Figure 3). The dental findings indicated widening of the periodontal space in the lateral dentition, suggesting that the condition may have been associated with occlusal trauma. Written informed consent was obtained from the subject for publication of this case report and the accompanying photographs, figures, and data.

### 2.2. Treatment Plan

Based on the above findings, the patient was diagnosed with occlusal trauma and an Angle Class I spaced dental arch associated with extensive moderate chronic periodontitis. The treatment objectives were resolution of the discrepancy, establishing appropriate anterior guidance by capturing the correct tooth axis inclinations for the maxillary and mandibular front teeth, and attenuation of the occlusal trauma. If there were stabilization of functional occlusion after use of a retainer and no progression of periodontal disease, we planned to perform palatal gingival grafting to the tooth cervix of the bilateral maxillary lateral incisors.

### 2.3. Treatment Progression

**Figure 5:**
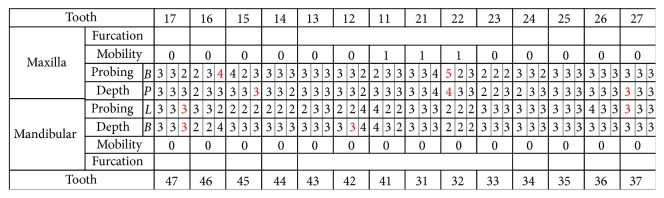
The periodontal examinations after periodontal treatment are shown. The red color indicates bleeding on probing.

A periodontal disease specialist treated the periodontal tissue before orthodontic treatment was commenced. We made the patient aware of the importance of her oral environment and explained the importance of being motivated for the ongoing oral management needed. Approximately 3 months later, there was improvement in the pocket depth, and the bleeding on probing and gingival swelling had resolved, indicating improvement of the periodontal disease (Figure 5). Given that the patient's awareness of the importance of maintaining her oral environment had also improved, we initiated active treatment.

For the active treatment, we used a Roth setup with a 0.022-inch slot bracket and started leveling using maxillary and mandibular 0.012-inch round nickel titanium wires. We then increased the wire size sequentially and were using a 016 × 022-inch stainless steel wire after 6 months. We then attached a hook between the maxillary and mandibular lateral incisors and canines and closed the gap using intermaxillary elastics to exert an extremely weak orthodontic force.

**Figure 6:**
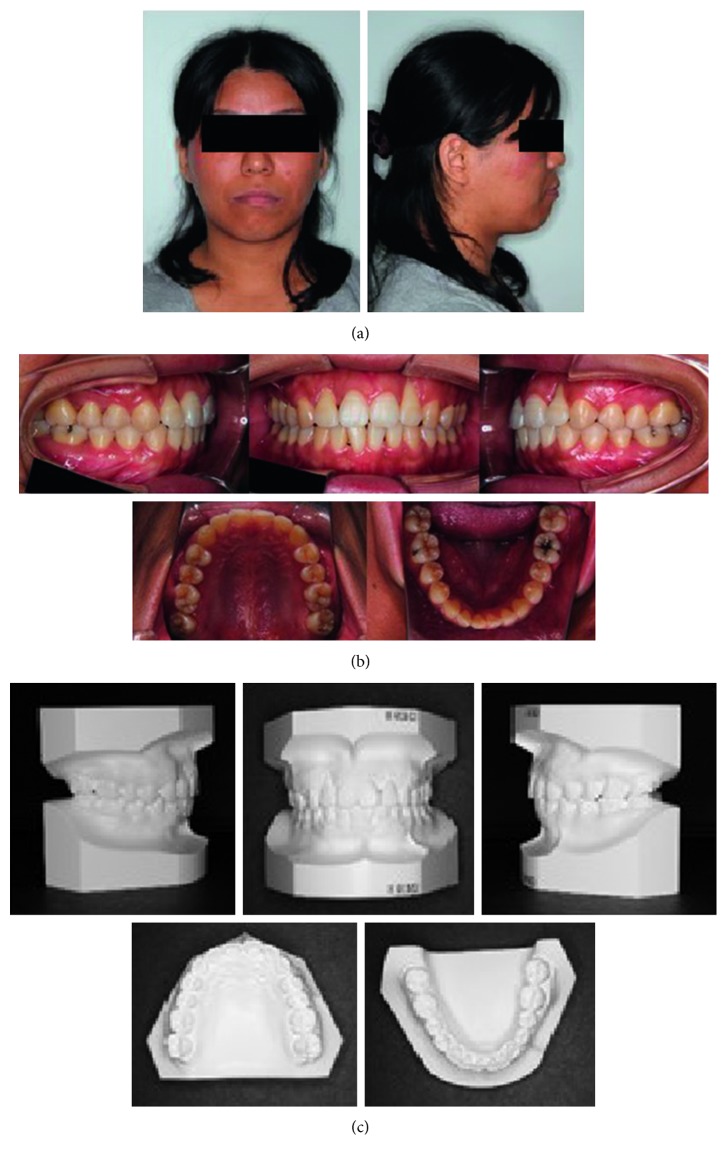
Post-orthodontic treatment facial (a) and intraoral photographs (b) and dental casts (c) are shown.

**Figure 7:**
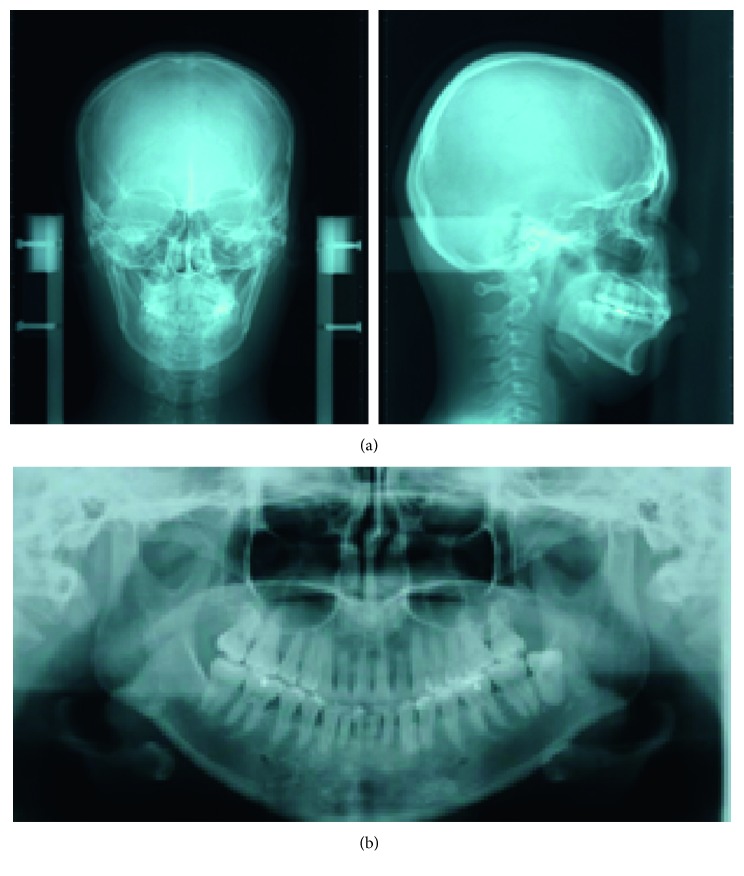
Post-orthodontic treatment cephalograms (a) and panoramic radiograph (b) are shown.

**Figure 8:**
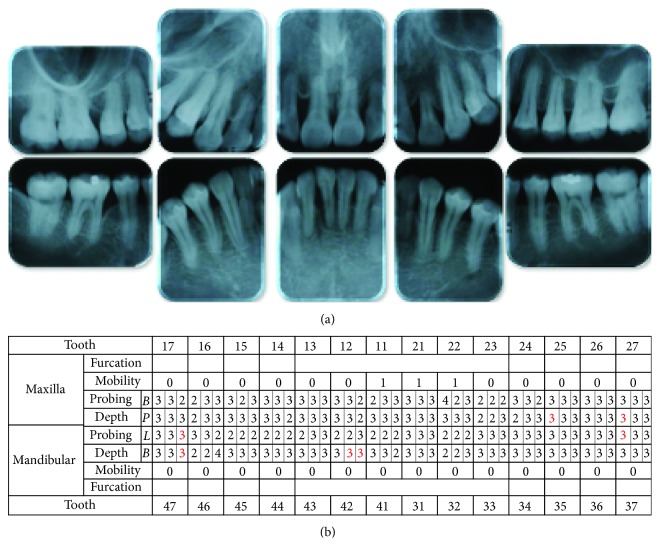
Post-orthodontic treatment dental radiographs (a) and periodontal examinations (b) are shown. The red color indicates bleeding on probing.

**Figure 9:**
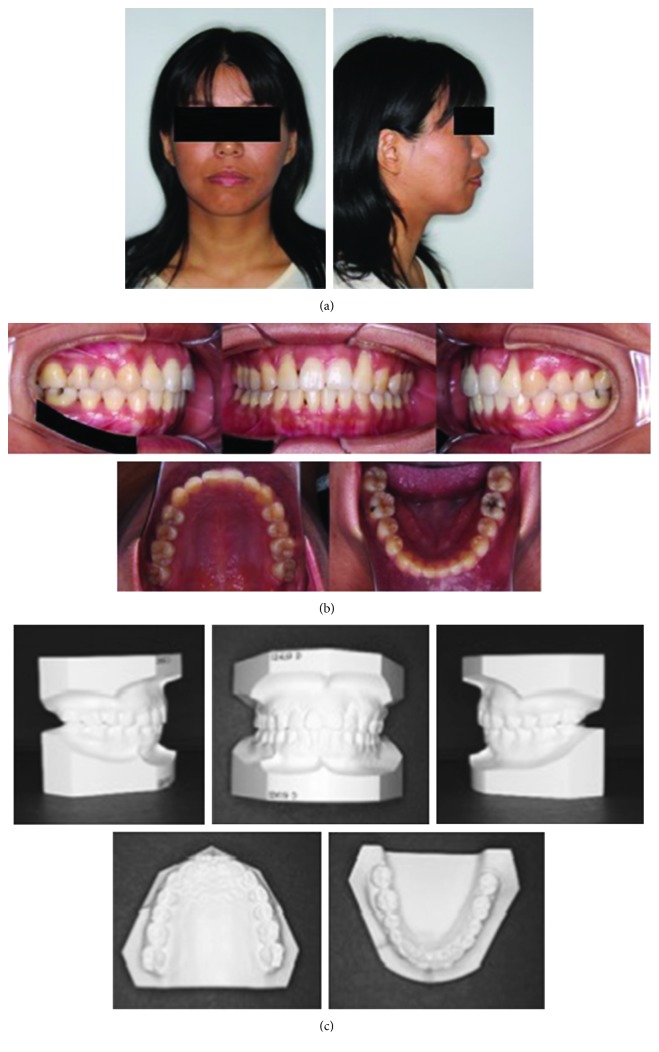
Postretention facial (a) and intraoral photographs (b) and dental casts (c) are shown.

**Figure 10:**
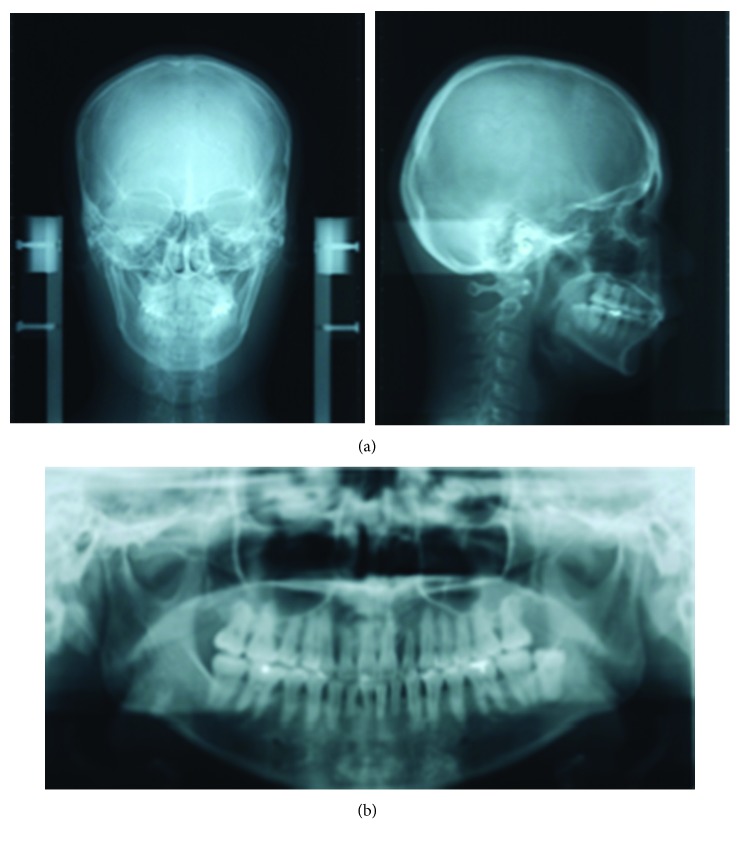
A post-retention cephalograms (a) and panoramic radiographs (b) are shown.

**Figure 11:**
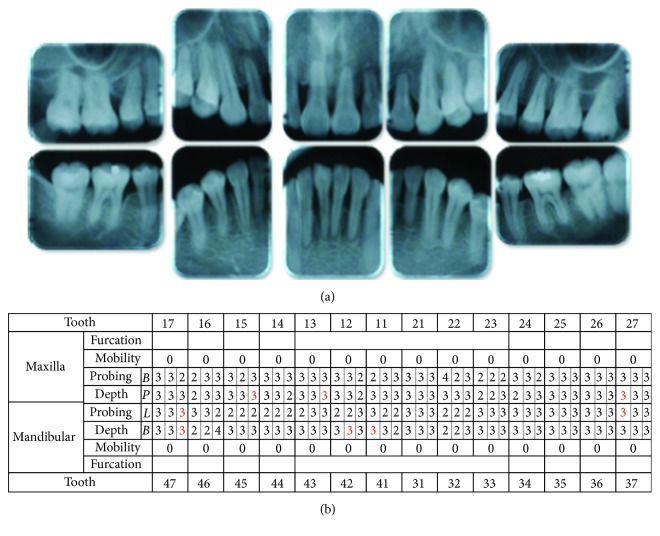
Post-retention dental radiographs (a) and periodontal examinations (b) are shown. The red color indicates bleeding on probing.

**Figure 12:**
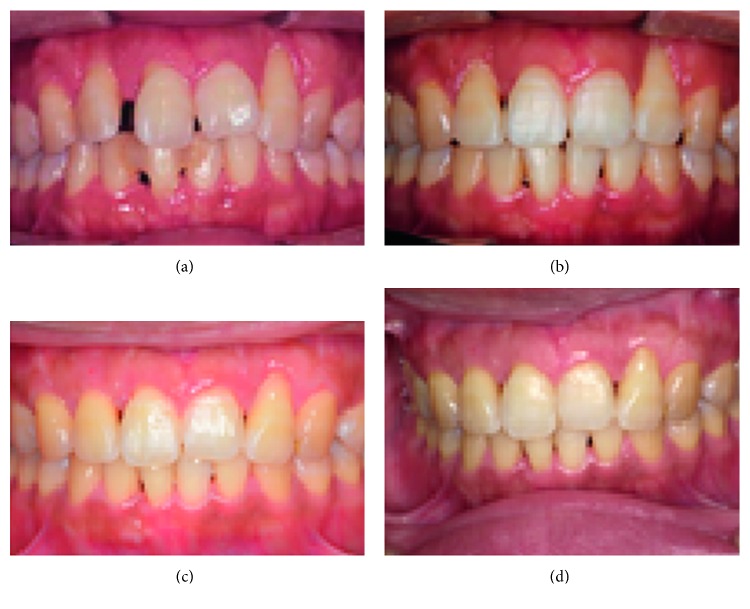
Frontal views of intraoral photographs are shown. (a) Before orthodontic treatment, (b) after orthodontic treatment, (c) at 6 months after periodontal surgical treatment, and (d) at 1 year 5 months after periodontal surgical treatment.

The BC showed strong functional contact with the right maxillary lateral dentition before starting active treatment, so construction of appropriate anterior guidance and molar spacing was considered for detailing. At the completion of active treatment, the patient started using a retainer after night-time parafunction was reassessed using the BC (Figure 4). The active treatment lasted 1 year and 8 months (Figures 6–8). A Begg-type retainer plate was used for both the mandible and maxilla. Two years after starting use of the retainers (Figures 9–11), the BC assessment was performed again, and the periodontal disease was reexamined (Figures 11 and 4). After the state of occlusion and the periodontal tissue were checked, the patient underwent coronally advanced flap repositioning surgery with a connective tissue graft for root coverage of the labial side of the tooth cervix of the bilateral maxillary lateral incisors using palatal mucosal connective tissue (Figure 12).

### 2.4. Treatment Results

In a photograph of the oral cavity taken after orthodontic treatment, the maxillary and mandibular spacing had closed, and a continuous and appropriate overbite and overjet were acquired. The findings indicated acquisition of good lateral incisor interdigitation (Figures 6 and 6). Panoramic findings indicated good parallelism of the roots of the teeth (Figure 7). Dental findings showed tooth root resorption of the left maxillary lateral incisors, but the lamina dura had become clearer. In addition, the widening of the right maxillary first molar periodontal space had disappeared, and bone regeneration was noted in the mesial area (Figure 8).

**Figure 13:**
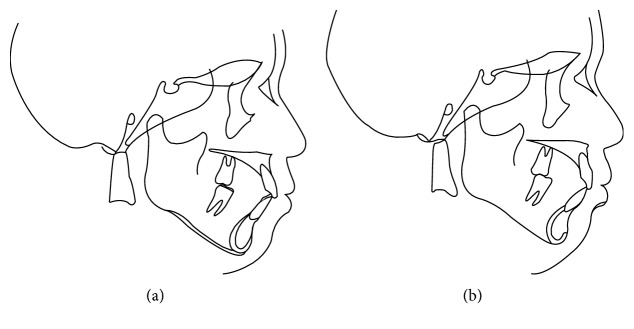
A superimposed cephalometric tracing is shown. (a) From pre-orthodontic treatment to post-orthodontic treatment; (b) from post-orthodontic treatment to postretention.

The BC findings after completion of treatment indicated weakening of the strong functional contact that was present in the right maxillary lateral dentition and front teeth (Figures 4 and 4). In the cephalometric superimposition (Figure 13), the mandible was slightly rotated clockwise, and the patient's profile was virtually unchanged. Dental findings showed slight elongation of the maxillary and mandibular molars, and the angles of the tooth axis inclinations of the maxillary front teeth had lessened (Figures 2 and 7, Table 1). After 2 years of using the retainer, when the BC assessment was performed again and periodontal disease was reexamined (Figures 4 and 9–11), there was no major change from that at the end of active treatment (Figures 7(a), 10, and 13(b) and Table 1). Therefore, the patient underwent coronally advanced flap repositioning surgery with a connective tissue graft for root coverage of the bilateral maxillary lateral incisors. Seventeen months after surgery, the patient had improved oral hygiene, had acquired esthetically good periodontal tissue, had stable functional occlusion, and was satisfied with the outcome (Figure 12).

## 3. Discussion

From the results of the cephalometric analysis, this patient did not have particularly major dental or skeletal malocclusion, but her facial type was convex, and the distance between the upper lip and the E-line was 2 mm and that from the lower lip to the E-line was 5 mm (Table 1). Therefore, we considered that extraction of the first premolar or the front teeth might improve her profile from lingual movement of the maxillary and mandibular front teeth. However, there was significant alveolar bone resorption in the areas of the maxillary and mandibular front teeth, so tooth extraction may have caused more significant loss of the supporting tissue, and the patient did not want tooth extraction to improve her profile. Therefore, treatment was commenced without tooth extraction.

In this patient, gingivitis, bleeding on probing, and tooth mobility were located in the maxillary and mandibular first molars and the maxillary and mandibular front teeth (Figures 3 and 3), so the patient was diagnosed with Angle Class I extensive moderate chronic periodontitis [4]. Further, there was widening of the periodontal space in the right maxillary lateral dentition, which is a characteristic of occlusal trauma. Therefore, this patient was investigated using the BC with the aim of visualizing and assessing the cause of the occlusal trauma (Figure 4), which is considered to be a factor exacerbating periodontitis [5, 6]. The results of that investigation confirmed that the location of functional contact in night-time parafunction correlated with the location of periodontal lesions on the chart. Thus, the aim of the orthodontic treatment was to close the spaced dental arch orthodontically and improve the tooth axes as well as to establish anterior guidance and reduce the functional lateral occlusal force of the lateral dentition as much as possible, thereby reducing occlusal trauma.

Orthodontic treatment for patients with periodontal disease not only exacerbates inflammation of the periodontal tissue with movement of the teeth, but may also promote resorption of diseased alveolar bone [8, 9]. Therefore, before starting active treatment with orthodontics, periodontal disease should be treated appropriately. This is essential to ensure ongoing management of the oral environment during orthodontic treatment. Before starting orthodontic treatment, we ensured that the inflammation was controlled by thorough periodontal treatment from a periodontal disease specialist, that the oral environment was stabilized, and that the patient thoroughly understood the importance of maintaining a stable oral environment (Figure 5). The orthodontic forces were set up to act continuously but as weakly as possible [10]. After leveling was complete, retraction on the maxillary and mandibular front teeth was performed using sliding mechanics with intermaxillary elastics. With detailing, appropriate step bends were performed as needed to achieve disclusion of the lateral dentition as much as possible during forward and lateral movement, and this was checked at each dental visit by moving the jaw in a gliding motion.

The BC was developed in 2006 as a simple way to observe parafunction during sleep [5]. The BC can observe occlusion at night-time to supplement information obtained from the articulator and/or chair side. Reports show that it is possible to observe the pattern, direction, and area of night-time grinding. Further, our own previous research [11] has shown a difference in contact aspects between occlusion observed on oral examination at the chair side and BC observations. We reported that there was less canine contact during night-time parafunction than when the patient was awake and increased contact on the working and nonworking sides of the molar region. The BC findings before removal of the device indicated that the gliding surfaces of the front teeth and the right lateral dentition were decreased in comparison with those before active orthodontic treatment (Figure 4). This was one of the factors in the decision to complete active treatment and start use of a retainer using a plate. In other words, at the completion of active treatment, the lateral occlusal force of the lateral dentition due to night-time parafunction and excess occlusive force from the front teeth had lessened. Because of the synergistic effect of treatment for periodontal disease, there was regeneration of the bone in the mesial right maxillary first molar region. We considered that it might also have worked effectively on recovery of the lamina dura in other locations of bone resorption. Animal models of induction of periodontal disease have shown that if traumatic occlusion is added, there is accelerated loss of attachment, and induction of alveolar bone osteoclasts is activated [12]. Based on this information, investigating this condition based on the results of an assessment of occlusion function with the BC and periodontal examination is considered effective in enabling occlusal treatment goal clarification through orthodontic treatment in cases of periodontal disease associated with occlusal trauma, as in the present case. We believe that an effective technique can be established as a research method for the relationship between periodontal findings and occlusion aspects.

After 2 years of using a retainer and after checking that there was no pathologic function contact on reassessment with the BC (Figure 4), we decided to perform periodontal surgical treatment for the bilateral maxillary lateral incisors. We were able to confirm that the bilateral lateral incisors had clearer lamina dura than that before surgery and that the periodontal tissue was stable (Figures 9 and 10). However, there was high-grade gingival recession, which was an esthetic failure. The depth of the gingival recession was more than 3 mm for both teeth, and the width of the keratinized tissue on the root apex side was 2 mm or greater. Therefore, we explained that it may not be possible to completely cover the root surface and performed coronally advanced flap repositioning surgery together with a connective tissue graft for root coverage [13]. The left lateral incisors had significantly more bone resorption than on the right side, which resulted in a lower coverage rate than on the right side. Root surface coverage has been reported in a systematic review by the American Academy of Periodontology in 2015 [14], and the report showed that predictability was high for Classes I and II in Miller's classification, but was low for Classes III and IV. Our patient had similar results, but was satisfied with the results, and after a year of recuperation, she continues to convalesce well (Figure 12).

## 4. Conclusion

Collaborating with a periodontal disease specialist to manage orthodontic treatment for patients with malocclusion associated with periodontal disease not only ensures a better oral environment, but is also considered effective in motivating patients to maintain a good oral environment. Visualization of night-time parafunction with the BC enables not only an examination of possible occlusal trauma by investigating the results together with the periodontal chart, but also clarification of treatment goals for occlusal reconstruction through orthodontic treatment.

## Figures and Tables

**Table 1:** Summary of the cephalometric findings.

Variables	Japanese norms (adult female)	Pretreatment	Posttreatment	Postretention
SNA (°)	82.3 ± 3.5	85	85	85
SNB (°)	78.9 ± 3.5	79	78	78
ANB (°)	3.4 ± 1.8	6	7	7
FMA (°)	28.8 ± 5.2	30	31	31
IMPA (°)	96.3 ± 5.8	99	102	102
U1 to SN (°)	104.5 ± 5.6	106	99	99
E-line to upper lip (mm)	−2.5 ± 1.90	2	2	2
E-line to lower lip (mm)	0.9 ± 1.90	5	5	5
